# Point-of-care ultrasound teaching in general practice and family medicine: EURACT position paper

**DOI:** 10.1080/13814788.2026.2666751

**Published:** 2026-05-22

**Authors:** Vesna Homar, Nele Roos Michels, Marek Kucera, Jachym Bednar, Yolanda Ortega, Camilla Vaskjær Aakjær Andersen, Martine Granek-Catarivas

**Affiliations:** a Department of Family medicine, University of Ljubljana, Ljubljana, Slovenia; b Interuniversity Centre for Education in Family Medicine, Leuven, Belgium; c Department of General Medicine, Comenius University Bratislava, Bratislava, Slovakia; d 1st Faculty of Medicine Charles University, Prague, Institute of General Practice, Prague, Czech Republic; e Department of medicine, University of Rovira i Virgili Reus, Tarragona, Spain; f Center for General Practice at Aalborg University, Aalborg, Denmark; g Department of Family Medicine, Clalit Health Services & Tel Aviv University, Tel Aviv, Israel

**Keywords:** Point-of-care ultrasound, primary care, education, family medicine, general practice, position statement

## Abstract

**Background:** Point-of-care ultrasound (POCUS) is an increasingly valuable tool in general practice/family medicine (GP/FM). This position paper from the European Academy of Teachers in General Practice/Family Medicine outlines recommendations for integrating POCUS education across the continuum of training: undergraduate, specialty, and continuing medical education.

**Recommendations:** POCUS should complement, not replace, the clinical examination. Key priorities include GP/FM-targeted curriculum development, early exposure during basic medical education, mandatory residency training, context-sensitive continuing professional development, train-the-trainer programmes, and GP/FM-led implementation.

**Discussion:** Integrating POCUS teaching across all stages of GP/FM education may support effective and sustainable adoption in primary care. Educational strategies should be adapted to local contexts and healthcare systems while maintaining a focus on clinically relevant and evidence-informed use.

**Conclusion:** EURACT recommends the longitudinal integration of POCUS education throughout GP/FM training and professional development. These principles aim to guide the effective, sustainable, and context-sensitive implementation of POCUS in primary care.

## KEY MESSAGES

- POCUS teaching in GP/FM should be integrated across basic medical education, specialty training, and continuing professional development.
- GP/FM targeted curriculum development, early exposure, and training during residency are essential for effective adoption.
- Context-sensitive continuing education and train-the-trainer programs support sustainable implementation.

## Introduction

Point-of-care ultrasound (POCUS) has increasingly become an integral diagnostic tool in general practice and family medicine (GP/FM) [1,2]. POCUS use in primary care has the potential to improve diagnostic accuracy, enhance physician confidence, positively impact patient outcomes, and is associated with high satisfaction among both patients and doctors [3]. It may also help reduce health inequalities and empower practitioners working in rural, remote, or under-resourced settings [4,5].

The European Academy of Teachers in General Practice and Family Medicine (EURACT) is a WONCA Europe network established in 1992, dedicated to fostering and maintaining high standards of education in GP/FM. It has more than 900 members from 40 different countries. EURACT has recognised early that POCUS addresses specific clinical needs and can improve patient outcomes in GP/FM. Based on the initiative of three countries (Czeck Republic, Slovenia and Israel) EURACT has established a POCUS task group in October 2023. The aim of this task group was to design EURACT recommendations on how to teach POCUS through all levels of medical training: 1) Basic Medical Education (BME) or undergraduate education, 2) Specialty Training (ST) and 3) Continuing Medical Education (CME), tailored to the unique needs of GP/FM and its healthcare contexts.

This position paper was drafted by the task group members from Slovenia, Israel, Spain, Slovakia, Czeck Republic, and Belgium through a collaborative process in the following two years. The development of the paper was highly impacted and justified by two important documents: 1) WONCA Europe’s position statement on the use of point-of-care ultrasound (POCUS) in primary care [3], published in 2024, and 2) the publication of an European core curriculum of point-of-care ultrasound examinations for frontline physicians in primary care, published in early 2025 [6]. Furthermore, the group reached out to the European Family Medicine Ultrasound Society (EFMUS) and European Federation of Societies for Ultrasound in Medicine and Biology (EFSUMB), which ensured a common perspective on the POCUS teaching and use in primary care and contributed to the development of this paper. This development approach mirrors consensus-oriented methodology, combining iterative multinational expert collaboration with literature-informed drafting, as was previously used within WONCA Europe networks [5]. A consensus and the adoption of this position paper was reached in autumn 2025.

## Recommendations for POCUS education in GP/FM

### POCUS curriculum in GP/FM context

- EURACT supports the development of a GP/FM-oriented POCUS curriculum framework that can be adapted to specific professional needs in different primary care settings [6–8].

POCUS is used for a wide variety of clinical indications that are specific to GP/FM and differ from other specialties [9]. A European Delphi consensus study defined 40 core POCUS indications for frontline physicians in primary care [6], and the American Association of Family Physicians suggested an even more extensive list [8]. However, it is neither feasible nor necessary to introduce teaching of all indications at once. A practical approach is to select a limited number of indications most relevant to the local setting, patient population (e.g., age, morbidity, cultural context), and available imaging facilities, including proximity and costs. Selecting up to 10 core context-specific indications has been suggested as a reasonable starting point, allowing learners to gain confidence and competence before expanding further [10].

- The curriculum should be evidence-based, reflecting best practices for POCUS use in primary care [9,11].

Effective POCUS use in GP/FM requires evidence-based education tailored specifically to the primary care context [6]. Physicians need to learn both general ultrasound skills and GP/FM-specific indications, with a clear understanding of the tool’s limitations. Documenting educational experiences, clinical impact, and patient outcomes can provide valuable insights. Sharing these findings through publications, conferences, or international collaborations helps strengthen the evidence base for POCUS in primary care, supports the development of best practice recommendations [1,6,12], and contributes to the growth of the global GP/FM POCUS community.

- Curricula at all levels of GP/FM training should promote the integration of POCUS into daily clinical practice as an extension of the clinical examination [9,11].

POCUS in GP/FM is performed by the physician who has taken the patient’s history, examined them clinically, and will plan further management [13]. Unlike comprehensive ultrasonography performed by radiologists, POCUS is focused, bedside, and provides rapid, dichotomous answers to specific clinical questions [13]. Training should emphasise that POCUS is an extension of the clinical examination, not a stand-alone or comprehensive ultrasonography exam. Defining a limited set of clinical indications helps ensure that POCUS is used appropriately - to confirm or refute diagnostic hypotheses derived from the patient’s history, presenting complaints, and physical examination [14].

### Basic medical education (BME)

 

- EURACT recognises the importance of early exposure to POCUS during BME [12,15]. Initial training may be delivered by other specialties to familiarise medical students with the principles of use and applications of POCUS.

European recommendations for ultrasound education for undergraduate medical students define extensive knowledge and skills, including basic foundations of POCUS [12]. A multidisciplinary POCUS training is recommended, involving various clinical specialties including GP/FM, so that students understand that the POCUS approach is universal, with differences arising only in the specific indications for each specialty [16]. General (non GP/FM specific) components of POCUS education include [8,9,12]: basic ultrasound physics, election of the appropriate probe imaging modes, sonographic appearance of structures, describing echogenicity, image optimisation—“knobology”, recognition of artefacts, probe orientation and scanning planes, probe handling movements and techniques, structured approach in the examination, image interpretation, documentation and quality assurance.

- In settings where POCUS is already integrated into GP/FM practice, students should be able to gain hands-on exposure to POCUS during the undergraduate GP/FM practice placements to appreciate its relevance to primary care.

During clinical training in GP/FM, students should have the opportunity to experience POCUS in everyday primary care, reinforcing its practical relevance. The limited number of GP/FM trainers with POCUS expertise highlights the potential value of incorporating peer learning among students in GP/FM settings.

### Specialty training

 

- Residents in GP/FM should receive dedicated training in POCUS as a mandatory part of their curriculum. This training should be tailored to the diagnostic and procedural needs of primary care [6]. It should focus on the integration of POCUS into everyday clinical practice as an extension of the clinical examination [17].

To facilitate the adoption of POCUS in GP/FM practice, it is important to integrate POCUS training into the core ST curriculum [18]. The content of a specialty training (ST) curriculum reflects the professional scope of GP/FM and delineates the core competencies required of GP/FM physicians [8]. During ST it is especially important to emphasise the impact of POCUS on medical decision-making, since trainees’ clinical reasoning is still developing. Incorporating POCUS in this way ensures that trainees acquire the necessary competencies as part of standard training, eliminating the need for additional post-certification or accreditation, and fostering a consistent standard of practice across GP/FM settings.

POCUS training should be embedded in the first half of the ST programme. During ST, trainees will have the opportunity to practice and refine their POCUS skills in the workplace, both in GP/FM settings and during hospital internships (e.g., internal medicine, surgery, paediatrics, emergency medicine). Peer learning should play an important role.

- GP/FM residency trainers represent a special subgroup that requires additional recognition and training to enable them to teach POCUS effectively in the GP/FM contexts.

As POCUS is a relatively new technology, trainees may develop proficiency before some of their GP/FM trainers, creating opportunities for reverse learning. Nevertheless, residency trainers should also have access to POCUS training to ensure effective supervision and support of their trainees.

### Continuous medical education

- All GP/FM physicians should receive training in POCUS that is tailored to the needs of their health care context. CME programmes should ensure that training is relevant and accessible so that doctors can effectively integrate POCUS into their clinical practice as an extension of the clinical examination [6,10].

CME learners are experienced clinicians with broad medical knowledge and clinical decision-making. Still, research consistently shows that GP/FM physicians who acquire POCUS skills benefit from improved diagnostic accuracy, increased confidence, enhanced patient outcomes, and greater job satisfaction [1,9,19].

CME programs should focus on GP/FM-specific indications, ensuring that training is relevant to primary care practice [6]. In a specific CME setting (e.g. country, region), it is reasonable to adopt a curriculum like that used in ST. Because the full POCUS curriculum cannot usually be covered in a single training, it should be divided into modular components, each addressing a subset of indications [6]. Stand-alone workshops alone do not anchor competency [7]. Instead, CME should be longitudinal, combining multiple training sessions with supervised hands-on practice in participants’ own GP/FM settings [10]. Programs should include formative follow-up to guide ongoing learning and summative assessment to confirm competence [10].

### Train-the-trainers

- EURACT supports the development of a train-the-trainers curriculum to facilitate the dissemination of GP/FM specific POCUS training [20].

Building a trainers’ scheme can be a considerable challenge in the early stages of implementation. POCUS trainers must be equipped to address the diverse learning needs for GP/FM setting, combining ultrasound competences, educational competences and GP/FM-specific field familiarity and clinical reasoning. This broadly corresponds to Level 2 of the European Federation of Societies for Ultrasound in Medicine and Biology (EFSUMB) training requirements [21].

Identifying potential POCUS trainers is a critical step. Suitable candidates may include early GP/FM POCUS adopters, graduates of POCUS training courses, and physicians with a particular interest in radiology or dual specialisations. These individuals should complete a tailored train-the-trainer program designed specifically for the GP/FM context. In addition, every training program should incorporate mechanisms to identify and nurture new trainers, creating a cascading model that steadily expands the trainers’ pool.

### Collaboration and support

- POCUS training in GP/FM can be initially supported by ultrasound or other specialists for consultation and guidance. GP/FM educators should lead the design and delivery of training programs, ensuring their alignment with the practical needs of primary care [11].

In early stages of POCUS adoption, the involvement of an experienced ultrasonography consultant or an experienced international GP/FM POCUS course provider is important to provide expert guidance and oversight. Trainers with qualifications equivalent to European Federation of Societies for Ultrasound in Medicine and Biology (EFSUMB) Level 3 would be appropriate [21]. As trainers gain proficiency and confidence, expert support can be provided through remote consultation, ensuring the continued quality of education and supervision [10].

All recommendations are summarised in Table 1.

**Table 1.** Recommendations for POCUS education in GP/FM.

1.POCUS Curriculum in GP/FM Context	•Support development of an adaptable GP/FM-specific POCUS curriculum framework
	•Ensure the curriculum is evidence-based and reflects best practices in primary care
	•Promote integration of POCUS into daily clinical practice as an extension of examination
2. Basic Medical Education (BME)	•Recognize the importance of early POCUS exposure during undergraduate training
	•Deliver initial POCUS teaching through other specialties to introduce principles and applications
	•Provide hands-on POCUS experience during GP/FM placements where already implemented
3. Specialty Training	•Include mandatory, tailored POCUS training in GP/FM residency focused on primary care needs and clinical integration
	•Provide additional recognition and training for GP/FM trainers to teach POCUS effectively
4. Continuous Medical Education (CME)	•Provide context-specific POCUS training for all GP/FM physicians that is relevant, accessible, and supports clinical integration
5. Train-the-Trainers	•Develop a train-the-trainers curriculum to expand GP/FM-specific POCUS teaching capacity
6. Collaboration and Support	Enable initial support from ultrasound/other specialists while ensuring GP/FM educators lead program design aligned with primary care needs

Box 1. Practical tips on POCUS education in general practice/family medicine (GP/FM).

**Figure:**
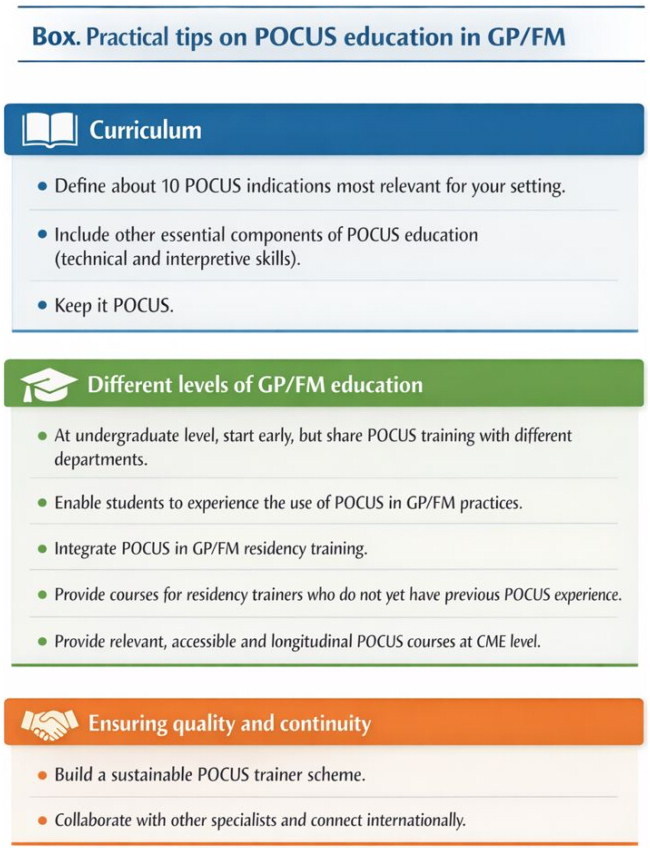

## Discussion

This position paper presents EURACT’s general recommendations on POCUS teaching in GP/FM. Practical tips are presented in Box 1. In this discussion, we address the impact of the recommendations on clinical practice, education and policy, including the feasibility and implementation of the suggested changes.

Evidence synthesised in recent WONCA Europe position papers suggests that POCUS in primary care may improve diagnostic accuracy, clinician confidence and patient outcomes, while potentially reducing referrals to secondary care and helping to mitigate health inequalities, particularly in rural or under-resourced settings [3]. These anticipated clinical benefits underpin the educational recommendations proposed here and support their relevance for countries where POCUS is already emerging in GP/FM practice [22–25].

Health systems and educational structures differ widely across Europe; therefore, implementation of these recommendations will inevitably be context-specific and phased. While integration across undergraduate, specialty training and continuing medical education appears feasible, successful uptake will require national and institutional curriculum adaptation and substantial investment in faculty development. EURACT’s advisory role is to provide a framework that may inform national training standards and policy discussions, rather than to mandate uniform adoption. From an economic perspective, existing studies suggest that POCUS use in primary care may be cost-effective through reductions in referrals and downstream investigations, although the available data remain limited and heterogeneous [1,2,26]. As this paper focuses primarily on educational strategy rather than service implementation, the direct costs of training, equipment acquisition and supervision warrant further formal evaluation in parallel with educational rollout.

Furthermore, access to ultrasound equipment and skilled POCUS course providers remains a key determinant of feasibility, and regionally adapted implementation strategies are likely to be required. Medicolegal frameworks also vary between jurisdictions and should be clarified at national level before widespread adoption. This process could be enhanced through collaborative relationships between specialised ultrasonography trainers (e.g. radiologists, cardiologists) and GP/FM trainers at all levels of POCUS education, starting with undergraduate training.

Future research should evaluate both educational outcomes and system-level impacts of POCUS education implementation in primary care.

## Conclusion

EURACT is committed to promoting the integration of POCUS into GP/FM education and practice, ensuring that general practitioners and family doctors are equipped with the knowledge and skills to use this valuable diagnostic tool for the benefit of patients. EURACT also encourages further research to evaluate educational strategies for effective and safe POCUS adoption in GP/FM clinical practice.
